# Effect of rapid palatal expansion on condylar displacement and TMJ space: A CBCT evaluation among pediatric population

**DOI:** 10.6026/973206300211098

**Published:** 2025-05-31

**Authors:** Yohan Verghese, Shruti R. Varshney, Tarunima Ghosh, Gitarani Hazarika Bora, Navdeep Kaur Shergill, Ruhi Sidhu, Yashwanth Kumar D.S.

**Affiliations:** 1 Department of Orthodontics, Saveetha Dental College, Chennai, Tamilnadu, India; 2 Department of Orthodontics and Dentofacial Orthodpedics, KD Dental College, Mathura, Uttar Pradesh, India; 3 Intern, Kalinga Institute of Dental Sciences, Kalinga Institute of Industrial Technology (KIIT), Deemed to be University, Patia, Bhubaneswar, Odisha, India; 4 Department of Orthodntics and Dentofacial Orthopedics, Regional Dental College, Guwahati, India; 5 Department of Oral Medicine and Radiology, Range Imaging Centre, Gurgaon, Haryana, India; 6 Department of Oral Medicine and Radiology, MNDAV Dental College and Research centre, Solan, Himachal Pradesh, India; 7 Department of Oral and Maxillofacial Surgery, College of Dental Sciences, Davanagere, Karnataka, India

**Keywords:** Rapid palatal expansion (RPE), condylar displacement, temporomandibular joint (TMJ) space, cone-beam computed tomography (CBCT), growing patients, orthodontics, skeletal changes, pediatric dentistry

## Abstract

The effect of Rapid palatal expansion (RPE) on condylar displacement and temporomandibular joint (TMJ) space using Cone-Beam Computed Tomography (CBCT) in a pediatric population is of interest. RPE with a Hyrax expander was done on 80 patients. Pre- and post-treatment CBCT scans were evaluated to measure condylar displacement (anterior-posterior and vertical) and TMJ joint spaces (anterior, posterior, superior and medial). RPE induces significant anterior and inferior condylar displacement and increases in TMJ joint spaces in growing individuals.

## Background:

Rapid Palatal Expansion (RPE) represents a cornerstone in contemporary orthodontics for the orthopaedic correction of transverse maxillary deficiencies, particularly in skeletally immature individuals [1, 2]. This modality involves the application of controlled lateral forces to the mid-palatal suture, leading to the separation of the maxillary halves and subsequent expansion of the basal bone. The biomechanical principle underlying RPE capitalizes on the plasticity of the craniofacial sutures during growth, enabling orthopaedic modifications that extend beyond mere dentoalveolar compensation [3, 4-5]. While the primary objective of RPE is to rectify maxillary constriction, its impact extends to adjacent craniofacial structures, including the nasomaxillary complex, pterygoid plates and notably, the temporomandibular joint (TMJ) [6, 7]. The TMJ, being a highly adaptive and load-bearing synovial articulation, is susceptible to positional and spatial alterations in response to orthopaedic forces transmitted through the maxillofacial skeleton [8]. It is hypothesized that the expansive forces generated during RPE may induce condylar repositioning-both anteroposteriorly and vertically-thereby modifying the intra-articular joint space. The interplay between RPE-induced skeletal remodelling and TMJ biomechanics remains an area of clinical significance, particularly in the context of growing patients whose craniofacial structures are in a dynamic state of development [9]. Therefore, it is of interest to evaluate the effect of Rapid Palatal Expansion on condylar displacement and TMJ intra-articular space using CBCT imaging in growing patients.

## Materials and Methods:

This prospective observational study was conducted in the Department of Orthodontics over a period of 18 months, from January 2023 to June 2024. The study population consisted of 80 growing patients, equally distributed between males (n=40) and females (n=40), aged between 8 and 14 years. The participants were selected based on specific inclusion criteria: patients in mixed or early permanent dentition exhibiting transverse maxillary constriction and deemed clinically suitable for Rapid Palatal Expansion (RPE). Patients with systemic conditions affecting bone metabolism, a history of temporomandibular joint (TMJ) disorders, craniofacial syndromes, traumatic injuries, or those who exhibited non-compliance during the course of treatment were excluded from the study. Baseline demographic and physical characteristics including age, gender, height (measured in centimetres), weight (in kilograms) and Body Mass Index (BMI) were documented. Systemic health status was recorded as healthy or non-healthy based on medical history and examination. Occlusal parameters evaluated included Angle's classification of molar relationship, midline deviation; overjet, overbite and palatal arch width at the level of the first molars and canines, all measured using CBCT imaging. All participants were treated using a Hyrax screw expansion appliance. The expansion protocol involved activating the appliance by 0.25 mm twice daily over a span of 14 days. Following the active phase, the appliance was kept in situ passively for a retention period of three months to allow for skeletal and periodontal stabilization. Cone-Beam Computed Tomography (CBCT) scans were obtained at two time points: prior to initiation of treatment (T0) and following the completion of the three-month retention phase (T1). All scans were captured using a standardized voxel size of 0.2 mm to ensure high-resolution imaging. The primary variables assessed from the CBCT included condylar position in both sagittal (anterior-posterior) and vertical planes, as well as intra-articular joint space measurements. These joint spaces included anterior joint space (AJS), posterior joint space (PJS), superior joint space (SJS) and medial joint space (MJS), measured in millimetres bilaterally. The CBCT images were evaluated using consistent reference planes and anatomical landmarks to maintain measurement reliability. All imaging data were interpreted by a calibrated examiner and intra-observer reliability was assessed prior to analysis. Statistical evaluations were performed using SPSS software (version 25.0), with significance levels set at p < 0.05.

## Results:

The study included a total of 80 growing patients, with an equal distribution of gender-40 males (50%) and 40 females (50%)-ensuring gender-based representativeness in the evaluation of the effects of Rapid Palatal Expansion (RPE). The mean age of the participants was 11.3 years with a standard deviation (SD) of ±1.7 years, indicating that the sample primarily consisted of children in the mid-growth period, an ideal stage for orthopedic intervention. In terms of physical parameters, the average height recorded was 142.8 cm ± 9.3 cm and the average weight was 38.6 kg ± 6.4 kg, both of which are within the expected range for the age group studied. The calculated mean Body Mass Index (BMI) was 18.7 ± 2.1, placing most participants within the normal weight range according to pediatric BMI classifications. These physical parameters confirm the overall health and growth status of the sample and provide a consistent baseline, minimizing systemic influences on craniofacial development and TMJ changes. By maintaining homogeneity in age, growth phase and general health, the study ensures that any observed changes in condylar displacement and TMJ space are attributable to the effects of RPE and not to external or unrelated biological variables (Table 1). The Table 2 demonstrates highly significant occlusal changes following Rapid Palatal Expansion (RPE). The intermolar width showed a marked increase from 28.6 mm to 34.2 mm, reflecting a mean gain of 5.6 mm, which is consistent with skeletal and dental expansion of the posterior maxilla. Similarly, the intercanine width increased from 21.3 mm to 26.1 mm, yielding an average increase of 4.8 mm, indicative of successful anterior expansion. Both parameters reached statistical significance with p-values < 0.001, confirming that the observed changes are not due to chance. These findings support the efficacy of the RPE protocol in producing transverse maxillary widening in growing patients. CBCT analysis revealed statistically significant displacement of the condyles following RPE treatment. The anterior (forward) displacement of the condyles averaged 1.2 mm, while the inferior (downward) displacement was 0.9 mm, both changes showing high statistical significance (p < 0.001).These findings indicate that RPE not only affects transverse skeletal dimensions but also induces measurable alterations in the sagittal and vertical positioning of the mandibular condyles. This is likely due to remodelling of the glenoid fossa and adaptation within the TMJ complex during skeletal expansion and stabilization phases. Clinically, this suggests that orthopaedic forces from RPE should be applied with consideration of their effects on TMJ positioning in growing patients (Table 3). Following Rapid Palatal Expansion (RPE), Cone-Beam Computed Tomography (CBCT) measurements revealed statistically significant increases in all assessed temporomandibular joint (TMJ) spaces. Specifically, the anterior joint space (AJS) increased by 0.4 mm (p = 0.003), the posterior joint space (PJS) by 0.4 mm (p = 0.001), the superior joint space (SJS) by 0.4 mm (p = 0.002) and the medial joint space (MJS) by 0.3 mm (p = 0.005). These dimensional changes suggest a consistent anterior and inferior repositioning of the mandibular condyles, resulting in an expanded joint capsule volume. This shift appears to be a physiological adaptation to the orthopedic forces exerted by the RPE appliance and represents functional remodeling within the TMJ complex during the critical growth period. Clinically, these findings underscore the need for vigilant monitoring of TMJ status throughout and following RPE therapy in growing individuals, ensuring that the expansion achieves skeletal correction without predisposing the joint to strain, instability, or dysfunction (Table 4). Age and gender demonstrated a significant influence on TMJ space changes (p < 0.05), suggesting that older age and gender may slightly impact joint space increases following Rapid Palatal Expansion (RPE). Additionally, height and molar relationship were significantly correlated with changes in joint spaces, particularly the anterior and posterior joint spaces (AJS and PJS), indicating that taller patients with specific molar relationships may experience more significant alterations in TMJ space. While BMI, weight, midline deviation, overbite and overjet showed minimal influence, some trends were observed. The palatal width, however, exhibited a significant association with increases in all joint spaces, highlighting the crucial role of skeletal changes in the success of RPE therapy (Table 5).

## Discussion:

The influence of Rapid Palatal Expansion (RPE) on temporomandibular joint (TMJ) space is shaped by a variety of skeletal, demographic and occlusal factors [10]. The patient's growth stage is a critical determinant, as younger individuals with more malleable bones respond more favorably to the orthopedic forces of RPE [11, 12]. Key parameters such as age, gender, height, molar relationship and palatal width each play distinct roles in the remodeling process within the TMJ [13]. Age and gender affect how the TMJ adapts to RPE, with older individuals potentially experiencing limited joint changes [14]. Height, often linked to craniofacial development, may correlate with more significant changes in TMJ space in taller patients, due to increased bone structure flexibility [15]. Molar relationships determine how forces are distributed across the maxilla, influencing the extent of joint space alterations [16]. While BMI and weight have a less pronounced effect, they still play a role in the overall adaptation due to their relationship with bone density and tissue flexibility [17]. Occlusal parameters, such as midline deviation, overbite and overjet, may influence force distribution, but their impact on TMJ space is relatively minor in this context [18]. Lastly, palatal width is directly related to changes in TMJ space, as palate expansion leads to the repositioning of maxillary bones, affecting joint dynamics. Understanding these parameters is essential to optimizing RPE therapy and ensuring functional TMJ health throughout treatment [19]. The study sample consisted of 80 growing patients, with a mean age of 11.3 years, an average height of 142.8 cm and an average weight of 38.6 kg. These values are consistent with the mid-growth phase, which is considered the ideal stage for orthopedic interventions like RPE. The findings are comparable to previous studies which suggest that the growth period between 7-14 years is optimal for RPE, as the maxilla is still malleable (Proffit *et al.*, 2007) [20]. The mean BMI of 18.7 ± 2.1 places the majority of participants in the normal weight range, which supports the study's focus on a generally healthy sample. This minimizes potential confounding factors related to systemic health, such as obesity or growth abnormalities, that could influence craniofacial development (Hancock *et al.* 2024) [21]. The analysis revealed significant increases in both intermolar and intercanine widths following RPE. The intermolar width increased by 5.6 mm and the intercanine width by 4.8 mm (p < 0.001). These changes are consistent with previous studies that report significant transverse maxillary widening as a result of RPE, particularly in growing individuals (Coloccia *et al.* 2021) [22]. The 5.6 mm increase in intermolar width is in alignment with studies by Cevidanes *et al.* (2010) [23], which documented an average increase of 5-7 mm in intermolar width following RPE in adolescents. Such significant changes in occlusal parameters reinforce the effectiveness of RPE in expanding the maxilla, leading to improved arch dimensions, as observed in previous literature (Ngan *et al.* 1997) [24]. The statistically significant changes further validate the clinical relevance of RPE in managing transverse maxillary deficiency in growing patients. Following RPE, CBCT analysis revealed significant anterior (1.2 mm) and inferior (0.9 mm) condylar displacement, both with p-values < 0.001. These findings suggest that RPE does not only induce transverse maxillary changes but also alters the sagittal and vertical positioning of the condyles. Similar results have been reported by Chung *et al.* (2004) [25], who found that RPE leads to anterior displacement of the condyles, likely due to the forces applied to the maxilla, which indirectly influence the TMJ. The anterior and inferior condylar displacement observed in this study indicates adaptive remodeling of the glenoid fossa, which is a key feature of the RPE process. These displacements are essential to consider clinically, as excessive condylar movements could lead to joint strain or dysfunction, though this study suggests that the observed displacements are within acceptable limits for growing patients.

This study found statistically significant increases in all measured TMJ spaces: anterior joint space (AJS) increased by 0.4 mm, posterior joint space (PJS) by 0.4 mm, superior joint space (SJS) by 0.4 mm and medial joint space (MJS) by 0.3 mm (p-values ranging from 0.001 to 0.005). The increases in joint space are consistent with the remodeling effects observed in the TMJ during orthopedic interventions like RPE, as reported in literature [13]. These findings suggest that RPE induces a functional remodeling of the TMJ complex, leading to an increase in joint space and a repositioning of the condyles. The results are in line with studies showing that maxillary expansion causes an adaptation of the TMJ, contributing to improved joint mobility without resulting in dysfunction [26]. The expansion of the joint capsule volume reflects the adaptive nature of the TMJ in response to the orthopedic forces exerted during RPE. In Table 5, the correlation between demographic, occlusal and physical parameters with TMJ space changes was assessed. Age, gender, height and molar relationship were found to have significant associations with changes in TMJ space, particularly the AJS and PJS. These findings corroborate those of studies by Kinzinger *et al.* (2006), who observed that older age and specific skeletal characteristics such as molar relationships influence the degree of TMJ space alterations following RPE [27]. The significant role of palatal width in influencing joint space changes (p = 0.01) aligns with the literature, which emphasizes the importance of skeletal changes during maxillary expansion. The palatal width is directly linked to the degree of expansion that can be achieved and its effect on TMJ space changes highlights the role of the skeletal framework in the success of RPE therapy. The clinical relevance of these findings lies in the importance of monitoring TMJ health during and after RPE treatment. While RPE is effective in expanding the maxilla and improving occlusion, the significant changes in TMJ space and condylar displacement highlight the need for careful management of the treatment forces to avoid potential long-term joint dysfunction. The correlation of demographic and skeletal parameters with TMJ space changes emphasizes the need for individualized treatment plans based on the patient's growth stage, skeletal morphology and occlusal characteristics. The study confirms that Rapid Palatal Expansion (RPE) significantly influences both condylar position and joint space morphology, with the observed anterior and inferior displacement supporting adaptive remodeling of the TMJ in response to orthopedic forces. The increase in joint space following expansion may indicate temporary decompression of the TMJ. However, the study's limitations include the short-term follow-up, concerns over CBCT radiation exposure and the absence of functional TMJ evaluation. Future perspectives suggest the need for longer-term studies incorporating functional TMJ assessments, correlation with masticatory muscle adaptation and integration with 3D occlusal and airway analysis to provide a more comprehensive understanding of the effects of RPE on the TMJ.

## Conclusion:

Rapid Palatal Expansion (RPE) in growing patients causes significant condylar repositioning and temporomandibular joint (TMJ) space alteration, underlining the importance of TMJ monitoring during orthopaedic maxillary expansion.

## Figures and Tables

**Table 1:** Demographic data (n = 80)

**Parameter**	**Mean ± SD**
Age (years)	11.3 ± 1.7
Height (cm)	142.8 ± 9.3
Weight (kg)	38.6 ± 6.4
BMI	18.7 ± 2.1
Gender	50% M / 50% F

**Table 2:** Occlusal changes

**Parameter**	**T0 Mean ± SD**	**T1 Mean ± SD**	**p-value**
Intermolar width (mm)	28.6 ± 1.9	34.2 ± 2.0	<0.001**
Intercanine width (mm)	21.3 ± 1.6	26.1 ± 1.5	<0.001**

**Table 3:** Condylar displacement (CBCT)

**Displacement Type**	**Mean Change (mm)**	**Direction**	**p-value**
Anterior	1.2 ± 0.5	Forward	<0.001**
Inferior	0.9 ± 0.4	Downward	<0.001**

**Table 4:** TMJ space alterations

**Joint Space**	**T0 (mm) ± SD**	**T1 (mm) ± SD**	**Mean Change**	**p-value**
AJS	2.3 ± 0.4	2.7 ± 0.5	+0.4 mm	0.003**
PJS	2.1 ± 0.3	2.5 ± 0.4	+0.4 mm	0.001**
SJS	2.0 ± 0.3	2.4 ± 0.4	+0.4 mm	0.002**
MJS	1.9 ± 0.3	2.2 ± 0.3	+0.3 mm	0.005**
(p< 0.01 indicates significance)

**Table 5:** Comparison of demographic, occlusal parameters with RPE-Induced TMJ Space Changes

**Parameter**	**AJS Change (mm)**	**PJS Change (mm)**	**SJS Change (mm)**	**MJS Change (mm)**	**p-value**
Age (years)	0.4 ± 0.2	0.4 ± 0.3	0.4 ± 0.3	0.3 ± 0.2	0.02**
Gender	0.4 ± 0.3	0.4 ± 0.3	0.4 ± 0.3	0.3 ± 0.2	0.05**
BMI	0.3 ± 0.2	0.3 ± 0.3	0.4 ± 0.3	0.3 ± 0.2	0.09
Height (cm)	0.3 ± 0.2	0.4 ± 0.3	0.4 ± 0.3	0.3 ± 0.2	0.04**
Weight (kg)	0.4 ± 0.3	0.4 ± 0.3	0.4 ± 0.3	0.3 ± 0.2	0.07
Molar Relationship	0.3 ± 0.2	0.3 ± 0.2	0.4 ± 0.3	0.3 ± 0.2	0.03**
Midline Deviation	0.3 ± 0.2	0.3 ± 0.2	0.4 ± 0.3	0.3 ± 0.2	0.06
Overjet (mm)	0.4 ± 0.3	0.4 ± 0.3	0.4 ± 0.3	0.3 ± 0.2	0.05**
Overbite (mm)	0.3 ± 0.2	0.3 ± 0.2	0.4 ± 0.3	0.3 ± 0.2	0.08
Palatal Width (mm)	0.4 ± 0.3	0.4 ± 0.3	0.4 ± 0.3	0.3 ± 0.2	0.01**
